# Commentary: Moderate exercise may prevent the development of severe forms of COVID-19, whereas high-intensity exercise may result in the opposite

**DOI:** 10.3389/fphys.2022.902739

**Published:** 2022-08-22

**Authors:** Metodija Kjertakov

**Affiliations:** Institute for Health and Sport, Victoria University, Melbourne, VIC, Australia

**Keywords:** physical exercise, high-intensity interval exercise, angiotensin-converting enzyme 2, immune system, SARS-CoV-2, COVID-19

There are two forms of angiotensin-converting enzyme 2 (ACE2) in the human body: membrane-bound (mACE2) form and soluble (sACE2) form (Xiao et al., 2020). Severe acute respiratory syndrome coronavirus 2 (SARS-CoV-2) was found to exploit mACE2 to enter the cells and cause coronavirus disease-2019 (COVID-19) (Hoffmann et al., 2020). In contrast, the exact role sACE2 plays in SARS-CoV-2 infection is still uncertain (Rahman et al., 2021).

A recently published paper by Hagiu (2021) discusses the potential of the intensity of exercise to impact the risk of developing severe forms of COVID-19 by modifying the behaviour of ACE2 activity. According to the author, moderate-intensity continuous exercise (MICE) may increase plasma sACE2 concentration, while high-intensity interval exercise (HIIE) may increase mACE2 expression. Furthermore, the author suggests that increased plasma concentration of sACE2 prevents the entry of SARS-CoV-2 into the cells, but increased mACE2 expression makes the cells more susceptible to the virus. Consequently, the author advances the hypothesis that whereas MICE should prevent the development of severe forms of COVID-19 *via* increased sACE2, HIIE could result in the opposite due to increased mACE2. This hypothesis is very interesting, but, as discussed below, it is too speculative and somewhat misleading.

First of all, Hagiu (2021) did not provide any scientific reference to support the idea that increased mACE2 expression may worsen the severity of COVID-19. In the COVID-19 literature, increased mACE2 level in human tissues has been proposed that could either increase disease severity due to higher viral load or contribute to the resolution of the disease by protecting the affected organs from the pro-inflammatory, pro-fibrotic, and pro-coagulant effects of circulating angiotensin II (Bourgonje et al., 2020; Chaudhry et al., 2020; Datta et al., 2020; Lanza et al., 2020; Wang et al., 2020). Unfortunately, the currently available evidence is insufficient to support or refute either of these ideas.

The paper in question also lacks an explanation why the author decided to base the part of the hypothesis related to HIIE on the idea that increased mACE2 expression could aggravate COVID-19 severity while ignoring the possibility that the same mACE2 response could be beneficial in a person infected by SARS-CoV-2 (Bourgonje et al., 2020; Chaudhry et al., 2020; Datta et al., 2020; Lanza et al., 2020; Wang et al., 2020). If one considers the latter possibility, an alternative hypothesis would be that an HIIE-induced increase in mACE2 expression could minimise the risk of developing severe COVID-19 complications. However, such a hypothesis would be as equally speculative as is the hypothesis that increased mACE2 expression associated with HIIE may worsen the severity of COVID-19. Even if the idea that increased mACE2 expression may increase COVID-19 severity is correct, no evidence exists that HIIE may increase the expression of mACE2 in the cell membrane of organs most affected by COVID-19, including the lung, liver, kidney, and gastrointestinal tract (Peiris et al., 2021). The findings of Klöting et al. (2020) presented by Hagiu (2021) that chronic HIIE increases ACE2 mRNA expression in myofibres of the trained muscle are not directly applicable to the organs more relevant for the outcome in COVID-19 patients. Still, using Klöting’s findings as support, Hagiu (2021) argues that performing HIIE leads to increased expression of mACE2 in vascular endothelial cells due to blood hypoxia. The notion that HIIE can cause a state of hypoxia, especially in active skeletal muscle, is not in question. However, the data from an *in vitro* experiment by Zhang et al. (2009) suggest that the hypoxia experienced during a typical HIIE session [∼20 min (Gillen et al., 2014)] is insufficient to cause an increased mACE2 expression in vascular endothelial cells. The researchers in that study (Zhang et al., 2009) had pulmonary artery smooth muscle cells exposed to 48-h hypoxia and found that ACE2 mRNA of the cells did not increase above the baseline level until after 12-h from the onset of the hypoxia.

Furthermore, it is not clear on what grounds Hagiu (2021) proposes that increased endogenous concentration of sACE2 may prevent the escalation of the symptoms of the disease. In the “Introduction” section of the paper, the author states: “*The soluble angiotensin-converting enzyme 2 (sACE2) form is present in both plasma and urine and it appears to play a role in preventing the virus from entering into the cell by competition with the transmembrane form (tACE2)*”, but he provides no reference to support the statement. Contrary to Hagiu’s idea about the potentially beneficial role of increased native sACE2 levels against COVID-19, three out of five clinical studies that measured plasma sACE2 in COVID-19 patients found an association between elevated plasma sACE2 levels and increased COVID-19 severity (Fagyas et al., 2022; Kragstrup et al., 2021; Reindl-Schwaighofer et al., 2021). The fourth study observed no correlation between plasma sACE2 and the disease presentation (Lundström et al., 2021), whereas the fifth study found that patients with the lowest circulating sACE2 had the most severe clinical outcome (Troyano et al., 2021). In light of those findings, it is currently not possible to predict what effects increased sACE2 concentration would have on the course of the disease in a person infected by SARS-CoV-2. Assuming that the hypothesis that increased sACE2 concentration can safeguard a person from severe COVID-19 complications is valid, then, based on the available evidence (Magalhães et al., 2020), and contrary to Hagiu’s proposal, HIIE would be more beneficial than MICE in inducing an increase in circulating sACE2 levels. Indeed, in the only available study that compared the effects of HIIE versus MICE on plasma concentrations of sACE2, Magalhães et al. (2020) found a statistically higher increase in sACE2 levels in response to the HIIE session in comparison to the MICE session. However, given the results of the studies cited above (Fagyas et al., 2022; Kragstrup et al., 2021; Lundström et al., 2021; Reindl-Schwaighofer et al., 2021; Troyano et al., 2021), the clinical significance of Magalhães’s findings in the context of prevention of COVID-19 and associated complications is unknown.

Hagiu (2021) also supplements his hypothesis by citing the study by Khammassi et al. (2020), who examined the chronic effects of MICE versus HIIE on immune function biomarkers in healthy young men. The researchers (Khammassi et al., 2020) showed that regular MICE increased leukocyte, lymphocyte, monocyte, and neutrophil counts, whereas HIIE had the opposite effect on these immune cells. Based on those findings, Hagiu (2021) argues that an additional mechanism by which HIIE may increase the susceptibility to SARS-CoV-2 infection is impaired immune function. However, that assumption could be misleading due to the following reasons. First, relying only on Khammassi’s findings when discussing the adaptations of the immune system to regular HIIE distorts the overall picture of the chronic effects of HIIE on immune function. Indeed, in a recent review of 36 studies, Souza et al. (2021) found that, although acute HIIE can cause short-term immunosuppression, long-term HIIE leads to favourable changes in immune function. Second, whether the decrease in the measured immune cells in response to HIIE in Khammassi’s study was of a magnitude sufficient to increase susceptibility to communicable diseases remains unknown, as the researchers did not attempt to investigate the potential relationship between those two factors. Elsewhere, impaired immune function resulting from a single HIIE session similar to those in the former study was not associated with an increased incidence of upper respiratory tract infections (Fahiman et al., 2001), suggesting that altered immune function reported following a typical HIIE session (Souza et al., 2021) may not be clinically relevant. Based on the currently available evidence, it appears only prolonged, fatiguing exercise can suppress immunity to the point where susceptibility to infection increases (Davis et al., 1997).

In summary, the idea that a person may develop severe forms of COVID-19 when infected by SARS-CoV-2 following HIIE due to increased mACE2 expression is unfounded. The same also applies to the idea that a MICE-induced increase in sACE2 concentration can prevent the development of severe forms of COVID-19 in a person infected by SARS-CoV-2. From the immunological point of view, there appears to be an agreement in the literature that MICE could be beneficial in preventing both susceptibility and severity of COVID-19 (Arazi et al., 2021; Da Silveira et al., 2021; Furtado et al., 2021; Laddu et al., 2021; Rahmati-Ahmadabad et al., 2020; Ranasinghe et al., 2020). There is also evidence that HIIE can induce immune-boosting effects in individuals accustomed to this form of exercise (Born et al., 2017; Monje et al., 2020). However, there is no evidence that a typical HIIE session (∼20 min) can cause immunosuppression post-exercise to a degree sufficient to increase the risk of infection. I feel it is necessary to bring this to the attention of readers because Hagiu’s hypothesis could discourage using a form of exercise whose benefits have been well-established in both health and disease (Sloth et al., 2013; Gillen et al., 2014; Wen et al., 2019; Maturana et al., 2020; Chen et al., 2021).
